# The role of cardiac lymphatic dysfunction in the progression of myocarditis

**DOI:** 10.3389/fcvm.2025.1659309

**Published:** 2025-10-27

**Authors:** Shreya Kurup, Daniel A. Hu, Tsutomu Kume

**Affiliations:** ^1^ Department of Medicine, Feinberg Cardiovascular and Renal Research Institute, Feinberg School of Medicine, Northwestern University, Chicago, IL, United States; ^2^ University of Illinois College of Medicine, Chicago, IL, United States; ^3^ Biological Sciences Division, Pritzker School of Medicine, The University of Chicago, Chicago, IL, United States

**Keywords:** lymphatic, lymphangiogenesis, myocardial edema, inflammation, myocarditis

## Abstract

Myocarditis, an inflammatory disease of the heart muscle, is a leading cause of sudden cardiac death in young adults and a major contributor to the development of dilated cardiomyopathy. Many studies highlight immune-mediated cardiomyocyte injury as a major contributor to myocarditis progression; however, myocardial edema may also play a significant role that has been overlooked. Beyond being a passive byproduct of inflammation, edema can mechanically stress the myocardium and create a proinflammatory microenvironment that may stimulate fibrosis, stiffen the myocardium, and impair cardiac function. Myocardial edema arises from an imbalance between vascular filtration and lymphatic clearance, often triggered by disrupted endothelial junctions that increase vascular permeability. The resulting accumulation of interstitial fluid fosters sustained inflammation, fibroblast activation, and extracellular matrix (ECM) remodeling. Furthermore, recent research highlights the therapeutic potential of targeting lymphatic regeneration to enhance edema resolution, attenuate inflammation, and limit fibrotic remodeling. This review examines the mechanistic pathways by which lymphatic dysfunction in myocarditis impairs lymphatic fluid clearance, focusing on the breakdown of lymphatic integrity, cytokine-mediated suppression of lymphangiogenesis, and maladaptive lymphatic remodeling. These processes contribute to adverse ventricular remodeling and dysfunction. Given that myocardial edema may be a key mediator for these pathological changes, we also discuss how emerging imaging techniques such as cardiac magnetic resonance (CMR) have enhanced the ability to detect and quantify edema, reinforcing its clinical relevance as both a diagnostic marker and prognostic indicator in myocarditis. Understanding the mechanistic pathways linking myocardial edema to pathology in myocarditis is promising for identifying novel therapeutic interventions.

## 1 Introduction

### 1.1 Overview of the lymphatic system

The lymphatic system is comprised of an extensive network of blind-ended vessels and intermittent lymph nodes that together ensure the unidirectional uptake and transport of fluid, macromolecules, and immune cells from the tissue interstitium back into the blood circulation (1, 2). The first point-of-contact for tissue fluid is the initial lymphatics, which are blind-ended sacs composed of a single layer of lymphatic endothelial cells (LECs) (3, 4). These LECs allow interstitial fluid to pass through and enter the lymphatic lumen (4, 5). The abluminal side of initial lymphatics is connected to the ECM via fibrillin-rich anchoring filaments (6, 7). Anchoring filaments maintain lymphatic capillary integrity and adjust permeability in response to interstitial fluid (IF) pressure, allowing greater fluid drainage when pressure rises (7, 8). High IF pressure creates tension in anchoring filaments, which pull apart overlapping endothelial flaps to permit lymph entry while preventing backflow into the interstitium (6, 7). These flaps are flanked by discontinuous button-like junctions that act as anchors, maintaining vessel integrity while allowing pores for fluid entry without junctional disassembly (9). Studies in mouse embryos have demonstrated the plasticity of these button-like junctions, as they can undergo button-to-zipper transformation under sustained inflammatory conditions, which can impair lymphatic vascular fluid uptake ability (10). After absorption through the initial lymphatics, lymphatic fluid flows through precollecting and collecting lymphatic vessels, ultimately draining into the bloodstream via the junction between the subclavian and jugular veins (6, 11). Precollecting vessels contain a mix of button-like and continuous zipper-like junctions that allow fluid containment, and are partially covered by muscle cells (10, 12, 13). Collecting vessels are LECs connected exclusively by zipper-like junctions, and these vessels are fully ensheathed by muscle cells and contain bicuspid valves that prevent lymph backflow, features meant to ensure proper lymph transport (6, 10). Lymphatic flow generally follows coordinated systolic and diastolic phases mainly regulated by external forces such as skeletal muscle contractions and heartbeats (14).

Proper lymphatic flow is essential for draining inflammatory mediators from peripheral tissue, while also transporting antigen-presenting cells and lymphocytes to lymph nodes, the key sites of antigen presentation and immune activation that regulate the strength and duration of inflammatory responses (15, 16). There are different ways that LECs can control the movement of immune cells. LECs can secrete several CC-type chemokines that attract different immune cells expressing their respective chemokine receptors (17, 18). Immune cells within the microenvironment, ranging from B cells to T cells to neutrophils, can also become drawn to LECs through adhesion molecules or pressure gradients that can pull them towards draining lymph nodes (19, 20). The passive removal of cytokines and antigen-presenting cells in lymph through lymph nodes also influences the duration of inflammatory responses, and lymphangiogenesis can modulate the speed of this process (21, 22). Conditions that impair proper lymphatic drainage, either through inefficient edema uptake by capillaries or poor transport by precollector or collector vessels, can cause an accumulation of edema that may prolong the initial inflammatory response (18). Proinflammatory cytokines such as TNF-α, together with infiltrating monocytes and macrophages, accumulate in regions of excess edema where they disrupt LEC junctions and increase vessel permeability (23). For example, edema-residing neutrophils can increase the presence of neutrophil elastase, which has been shown to degrade EMILIN1, an ECM glycoprotein in anchoring filaments of lymphatic capillaries (24). Defects in anchoring filament action can harm lymphatic vessel drainage function and result in further accumulation of edema (24, 25). Chronic inflammation can also transform the normally discontinuous cell-cell junction of initial lymphatics into a more continuous, closed form, which reduces the vessels' ability to clear lymph and its inflammatory components from the tissue (10). Additionally, arachidonic acid products like prostaglandins have been shown to reduce pumping action and the amplitude of lymphangion contractions in lymphoedema animal models (26). Combined with studies showing that anti-TNF therapy restores lymphatic contractions and vessel integrity in TNF-transgenic mice, these findings highlight that inflammatory signaling can impair lymphatic drainage through structural and functional mechanisms, creating a positive feedback loop of edema formation and sustained inflammation (23).

### 1.2 Overview of the cardiac lymphatic vasculature

The two main mechanisms for the development of cardiac lymphatic vessels are through lymphangiogenesis, the sprouting of new lymphatic vessels from pre-existing ones, and lymphvasculogenesis, the formation of lymphatic vessels through the merging of lymphatic endothelial precursor cells (27–29). Lymphvasculogenesis is important for the initial formation of the lymphatic vessels during development, whereas lymphangiogenisis drives the expansion of the lymphatic network and supports remodeling and repair in pathologic states. It is well-established that the lymphatic vasculature forms during embryogenesis following the development of the major vascular structures, the dorsal aorta and the cardinal vein, which arise from mesenchymal progenitors known as angioblasts (11, 30). However, the origin of cardiac lymphatics in particular has been debated; multiple cellular sources, both venous and non-venous, have been suggested to contribute to the development of the initial lymphatic structures in the heart (31). While the paraxial mesoderm has been described extensively as the predominant source of cardiac LEC precursors, recent studies suggest that additional populations may originate from the hemogenic endothelium, particularly from the yolk sac, and the second heart field, a cluster of arterial and sub-mesothelial cells located at the base of the pulmonary artery (31, 32). LEC precursors may also be regionally restricted: cardinal vein-derived LECs predominantly populate the dorsal side of the heart, whereas second heart field-derived LECs source the ventral side, including Islet-1-expressing non-venous progenitors contributing to LECs around the outflow tract and ventricles (32, 33).

Studies on murine hearts have characterized the developmental milestones of the cardiac lymphatics. The first cardiac LECs have demonstrated to emerge from the cardinal vein around embryonic day 12.5 from the extracardiac region near the ventral outflow region (31). As development progresses, lymphatic vessels appear on the ventricular surface, sprouting from areas close to the sinus venosus on the dorsal side (31). Lymphatic growth continues with dorsal vessels extending downward from the inflow region and smaller vessels emerging between the atria. The lymphatic vasculature continues to extend from both the ventral and dorsal regions to the apex of the heart, eventually forming a more branched network that covers the majority of the subepicardial layer by postnatal day 15.

Cardiac lymphatic development begins mid-gestation with the emergence of prospero homeobox protein 1 (PROX1) expressing LECs derived from endothelial progenitors originating from the paraxial mesoderm (34). For LEC specification to occur, the transcription factors SOX18 and COUP-TF2 bind to the regulatory region of *Prox1*, allowing PROX1 transcription and suppressing arterial differentiation (35, 36). However, this process is also regulated by secreted factors such as Wnt5b and Bmp2b, which can indirectly influence the rate at which endothelial cells express PROX1 (37–39). LEC identity is maintained through a positive feedback loop between PROX1 and vascular endothelial growth factor (VEGF)-C (40). Lymphatic structures can be identified during early development by their expression of lymphatic vessel endothelial hyaluronic acid receptor 1 (LYVE1) (18).

Different species have different distributions of lymphatic vessels in the heart; while mice have a greater density of lymphatics in the subepicardial than the subendocardial region, humans have a continuous plexus of lymphatic vessels that span the myocardial, subepicardial, and subendocardial areas (41, 42). In humans, lymph flow moves from capillaries in the subendocardium and myocardium and merges into collecting vessels in the subepicardium (43). When comparing cardiac regions, the ventricles contain more lymphatic vessels than the atria (44). Diastolic ventricular filling increases chamber pressure, propelling the movement of lymph from the subendocardial to the myocardial lymphatics (43, 45). The ventricular contractions that occur during systole are important for pushing lymph from the myocardium to the subepicardial lymphatics. The critical role of proper lymphatic flow in maintaining myocardial function has been demonstrated in murine models of myocardial ischemia, where impaired drainage has led to fluid accumulation, subsequent fibrosis, and the recruitment of neutrophils, monocytes, and macrophages (46). In humans, studies of acute decompensated heart failure demonstrate that the resolution of myocardial interstitial edema, as reflected through global left ventricular T2 values, improved cardiac hemodynamics, underscoring the importance of lymphatic clearance for maintaining myocardial function (47).

### 1.3 Consequences of edema on cardiac structure and function

Theories for how edema passes in and out of the vasculature have constantly been revised. The traditional idea follows Starling's law, which highlights that hydrostatic pressure is the root cause for fluid loss from plasma, and the osmotic gradient from macromolecules counteracts fluid loss from the vasculature (48, 49). Edema occurs when the balance between blood filtration and lymphatic reabsorption is disrupted, either because lymphatic capillaries cannot keep pace with fluid accumulation or because heightened vascular permeability overwhelms the lymphatic drainage capacity (50, 51). Together, these processes underscore that cardiac fluid homeostasis depends on the delicate balance between vascular filtration and lymphatic drainage. When this equilibrium is disrupted, persistent myocardial edema can develop, driving pathological remodeling and inflammation. Understanding these and other potentially interconnected mechanisms is crucial for elucidating the full impact of lymphatic dysfunction on cardiac health.

In the healthy heart, small amounts of fluid continuously filter out of the cardiac capillaries into the interstitium and are cleared by the cardiac lymphatic system, preventing fluid accumulation (52). One hallmark of myocarditis is that it presents with excessive myocardial edema, which directly impairs both the structure and function of the heart (53, 54). In fact, even a 3% increase in myocardial water content results in a ∼30% reduction in the heart's ability to maintain cardiac output (55). Myocardial edema is a common diagnostic feature of myocarditis and is frequently localized to the midwall and subepicardial layers on cardiac magnetic resonance (CMR) imaging, as seen through T2-based imaging (56, 57). While CMR-detected edema has not been shown to independently predict outcomes, myocardial edema contributes to disease progression by promoting inflammation, activating fibroblasts, and driving fibrotic remodeling (58–60). Myocardial edema contributes to ECM remodeling through both molecular and mechanical pathways. Alterations in interstitial pressure are sensed by fibroblasts, promoting their differentiation into a profibrogenic myofibroblast phenotype and increasing collagen production and altering the ECM composition (61–63). Concurrently, elevated hydrostatic pressure disrupts endothelial cell-matrix attachments, upregulating fibronectin fibers and receptor expression to initiate early ECM remodeling (64, 65). Collectively, these processes underscore the central role of myocardial edema in driving fibrotic remodeling.

The presence of myocardial edema can exacerbate electrical instability through inflammation-driven tissue remodeling, promoting conduction abnormalities that increase susceptibility to atrial fibrillation and ventricular tachycardia, which are common arrhythmic complications in patients with myocarditis (66–69). The duration of myocardial edema can also impair the heart in distinct ways (70). Acute myocardial edema physically stresses the myocardium and impairs the clearance of pro-inflammatory factors, prolonging the local presence of cytokines and chemokines and amplifying immune cell recruitment (71). Infiltrating leukocytes can induce cardiomyocyte death, releasing damage-associated molecular patterns that activate the complement cascade (mainly through the alternate pathway) and trigger endothelial cell activation, which furthers the production of reactive oxygen species and proinflammatory cytokines (71). The resulting inflammatory and oxidative stress disrupts interendothelial junctions, promoting additional fluid and immune cell accumulation, and thereby perpetuating local immune activation, extending tissue injury, and reducing left ventricular compliance (70, 71). During chronic myocardial edema, the changes in interstitial flow and pressure activate cardiac fibroblasts, promoting collagen production and myofibroblast differentiation through angiotensin II/AT1 and syndecan-4/NFAT signaling pathways (72). These processes not only enhance ECM stiffness by increasing collagen cross-linking via lysyl oxidase, but also contribute to the accumulation of collagen types I and III (73, 74). Additionally, chronic myocardial edema has been shown to further stabilize collagen fibers by upregulating prolyl 4-hydroxylase activity, increasing collagen resistance to degradation and amplifying fibrotic remodeling (51). These processes directly increase left ventricular myocardial stiffness, which may help explain why myocarditis often progresses to extensive myocardial scarring, left ventricular remodeling, and ultimately the development of dilated cardiomyopathy (75). The alterations in ECM composition also increase oxygen diffusion distances by forcing oxygen to traverse dense, excessive collagen fibers between capillaries and cardiomyocytes, ultimately impairing cardiac function and exacerbating ischemic injury (55).

## 2 Mechanistic pathways linking myocarditis to lymphatic dysfunction and edema

### 2.1 Pathophysiology of myocarditis

**Figure 1:**
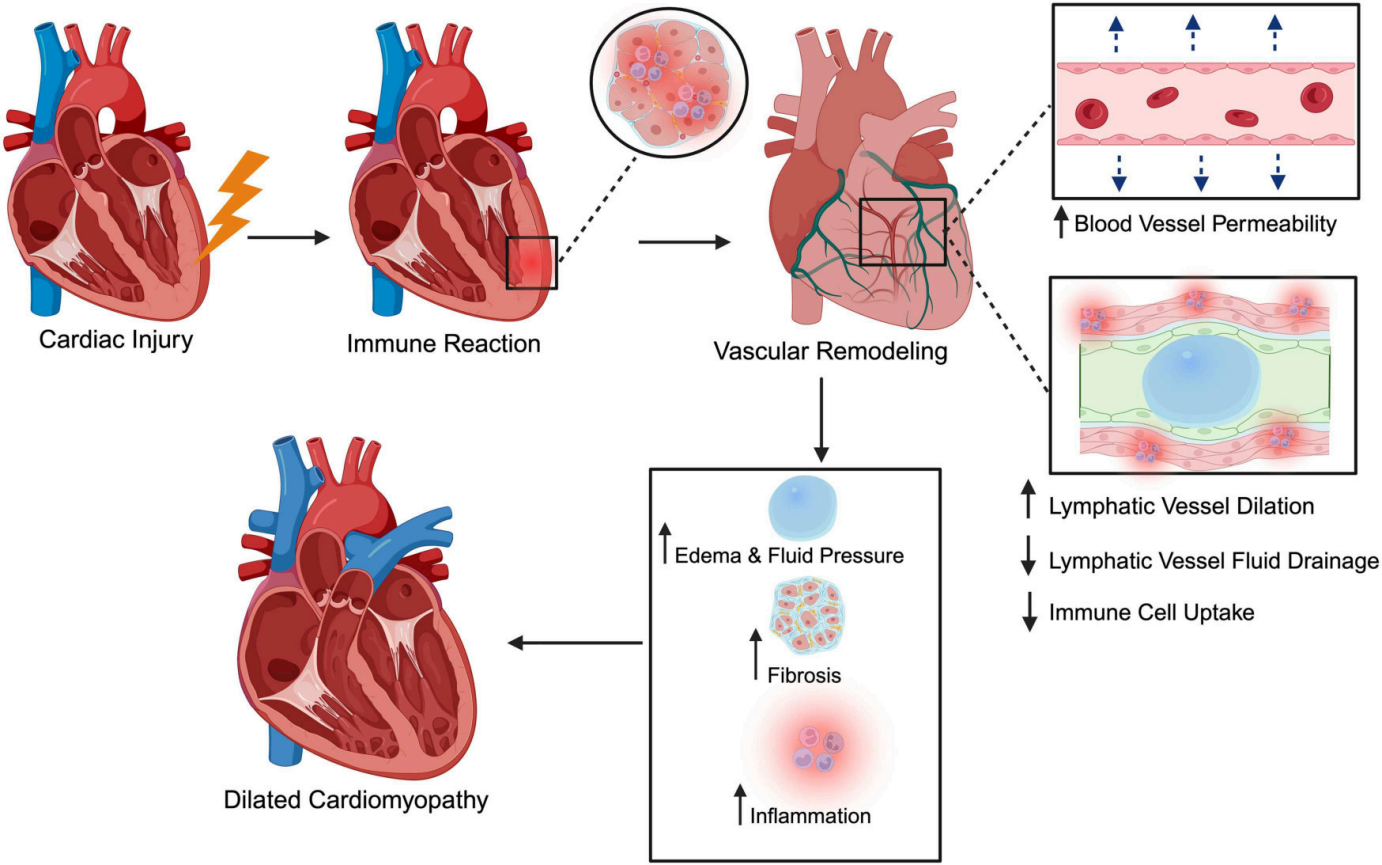
Schematic of myocarditis progression from acute injury to chronic disease. A variety of infectious and non-infectious triggers can cause initial cardiac injury. This leads to an immune and inflammatory response, causing vascular remodeling characterized by increased blood vessel permeability and disruption to lymphatic flow, impairing fluid drainage and immune cell trafficking. The resulting accumulation of interstitial fluid and inflammatory factors promotes fibrosis and ventricular dilation, ultimately progressing to conditions such as dilated cardiomyopathy. The figure was created using the illustration software on BioRender.com.

Myocarditis is an inflammatory disease of the heart muscle characterized by leukocyte infiltration into the myocardium with associated cardiomyocyte necrosis that is not due to ischemic injury (76). It can be triggered by a wide range of insults, from infections ranging in nature between bacterial, fungal, parasitic, and viral, to non-infectious immune-mediated causes such as allergens and autoantigens such as giant cell myocarditis and systemic autoimmune disease (77). Regardless of the inciting cause, the pathophysiological cascade typically involves an initial injury to cardiac myocytes followed by an immune reaction (Figure 1). In virus-induced cases, for instance, direct viral infection of cardiomyocytes and myocardial antigen-release activate the innate and adaptive immune responses, leading to myocyte necrosis, inflammatory cell infiltration, and tissue edema (78). This acute inflammatory phase may be self-limited; in many patients, the immune response is downregulated, and the myocardium gradually recovers without lasting damage. However, in a subset of cases, the inflammation persists, causing ongoing myocyte injury and downstream remodeling of the ventricular architecture (79). Over time, persistent inflammatory injury can lead to excessive ECM deposition and chamber dilation, ultimately progressing to dilated cardiomyopathy and chronic heart failure (80). Notably, myocarditis is recognized as one of the major antecedents of idiopathic dilated cardiomyopathy (DCM), with up to 30% of DCM cases attributed to previous myocarditis (77, 81). It is also an important cause of sudden cardiac death in young adults (81, 82), highlighting its clinical significance.

While the significant roles of the blood vasculature in myocarditis (increased capillary permeability, endothelial dysfunction, and inflammatory cell extravasation) have been well-established, the involvement of the cardiac lymphatic system remains less characterized in the pathophysiology of myocarditis (79, 83). During myocarditis, immune-mediated injury extends beyond blood microvasculature to the lymphatic network, unleashing a cascade that converts transient inflammation into persistent, edema-driven damage. In this section, we will outline three overarching processes which contribute significantly to this process: heightened lymphatic permeability; pump paralysis, in which cytokines such as IL-1β and TNF-α blunt intrinsic lymphangion contractility; and maladaptive remodeling, marked by capillary dilation, pre-collector narrowing, and loss of anchoring filaments that tether vessels to the ECM (Table 1). The net result is stalled clearance of interstitial fluid and pro-inflammatory mediators, escalating interstitial pressure, mechanically activating fibroblasts, and accelerating the transition to fibrosis and DCM. The subsections that follow will examine each step in greater detail.

**Table 1:** Mechanistic pathways by which myocarditis disrupts cardiac lymphatics and drives myocardial edema.

Pathway/Mechanism	Representative events	Key mediators/molecules	Functional consequences	Ref.
1. Barrier failure				
Anchoring filament injury	Collapse or poor opening of initial lymphatics	Collagen and GAG-degrading enzymes	Impaired fluid entry	(86–88)
2. Pump paralysis				
Cytokine suppression of contractility	COX-2/PGE_2_-driven relaxation of lymphatic pumping	IL-1β, TNF-α, COX-2, PGE_2_	*↓* Frequency & force of lymphangion contractions; stagnant lymph	(93, 94)
Network transport mismatch	Capillary dilatation with pre-collector constriction; thoracic duct back pressure	VEGF-C–induced capillary growth; inflammation-driven caliber loss	Engorged capillaries with downstream bottleneck; interstitial fluid stasis	(95–98)
3. Lymphangiogenic remodeling				
Adaptive	VEGF-C/VEGFR-3– driven sprouting with mature collectors	Macrophage VEGF-C, VEGF-D, IL-7 *→* VEGFR-3	Restored drainage, reduced inflamma- tion, functional recovery	(100–102)
Maladaptive	Delayed capillary-only growth; lack of collectors	Sub-threshold VEGF- C/D, persistent cytokines	Persistent edema *→* fibroblast activation, fibrosis, DCM progression	(101, 102)

#### 2.1.1 Structural damage and permeability shifts

##### Anchoring-filament disruption and weakened intercellular junctions

**Figure 2:**
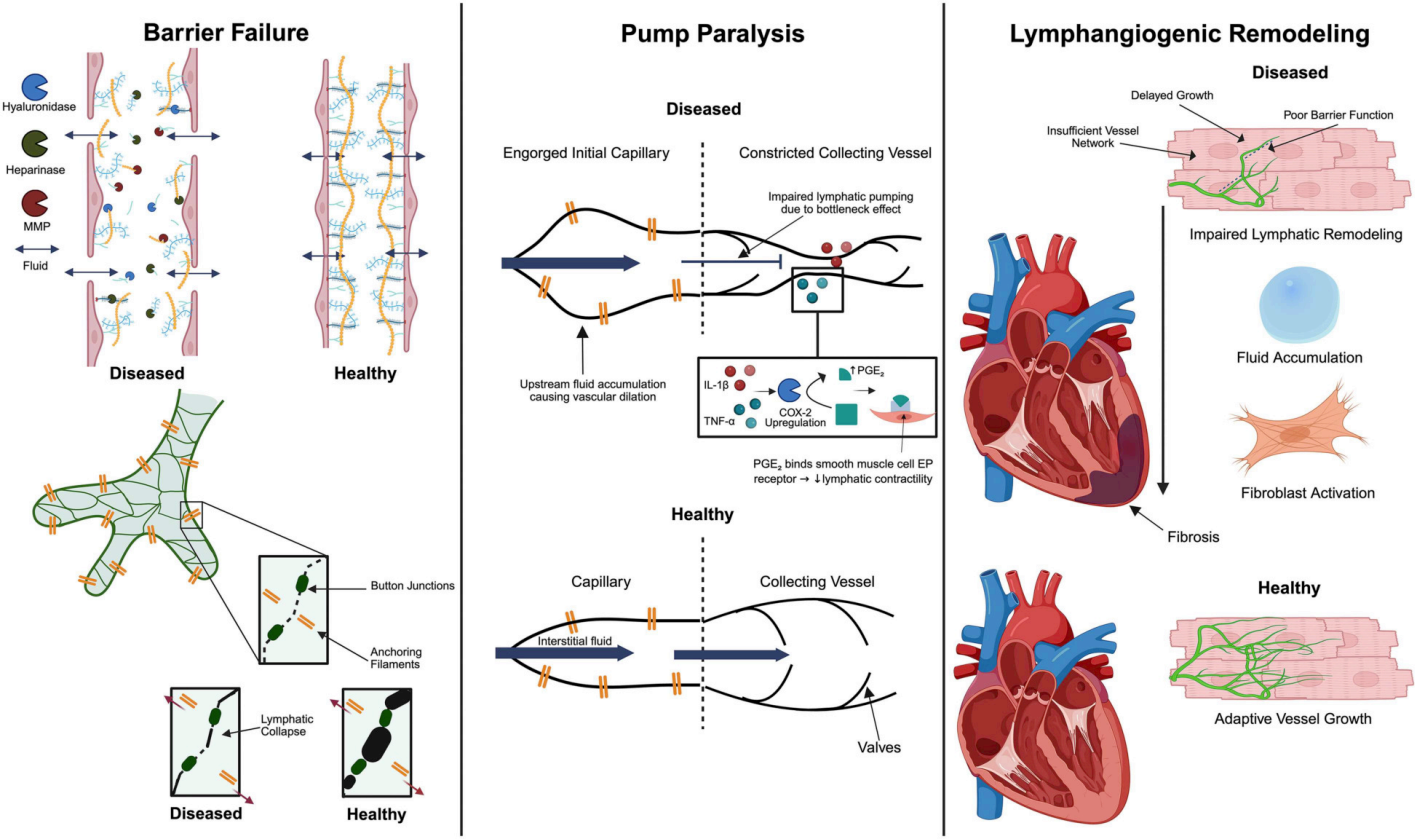
Key mechanisms of cardiac lymphatic dysfunction in myocarditis. There are three interconnected contributors: anchoring filament disruption that compromises lymphatic vessel integrity, inefficient lymphatic pumping due to the constriction of collecting vessels by inflammatory cytokines and subsequent dilation of downstream capillaries, and dysregulated lymphangiogenic remodeling. Together, these changes impair lymphatic clearance, allowing myocardial edema and inflammation to persist and promoting fibrotic remodeling. The figure was created using the illustration software on BioRender.com.

Inflammatory remodeling of the ECM undermines lymphatic integrity. Initial lymphatic capillaries are suspended by slender, fibrillin-rich anchoring filaments (∼10 nm elastin-like strands) spanning from the abluminal edges of lymphatic endothelial cells to the surrounding cardiac ECM. During edema, rising interstitial tension pulls the lymphatic walls open (84, 85). These filaments normally maintain lymphatic patency through the exertion of tensile forces, facilitating lymphatic flow. During acute myocarditis, anchoring filaments are disrupted not only by proteolytic enzymes, but also by tissue swelling, leading to lymphatic dysfunction. Specifically, edema-induced distortion of the interstitium and activation of collagen- and GAG-degrading enzymes have been shown to negatively impact anchoring filaments, compromising lymphatic lumen patency (86). Taken together, while acute lymphatic obstruction primarily results in edema, chronic obstruction can lead to interstitial fibrosis and remodeling of the ECM. Oxidative stress can also injure LECs directly by disrupting LEC junctions, compounding the loss of barrier function (87). These structural derangements are key contributors to lymphatic dysfunction in myocarditis (Figure 2). Leaky and structurally unsound lymphatic vessels cannot contain or transport fluid effectively, establishing the substrate for interstitial fluid accumulation (3). This allows protein-rich fluids and cells to flood the myocardium, initiating edema that feeds forward into further inflammation (88).

#### 2.1.2 Functional impairment of lymphatic pumping

While structural breach of lymphatic integrity initiates myocardial edema, the persistence of fluid overload is largely driven by a second hit: failure of the lymphatic “pump” itself. Cardiac lymphatics rely on the periodic motion of cardiac contraction to passively propel lymph flow (89). In myocarditis, an intense cytokine milieu, altered wall stress, and hemodynamic congestion converge to blunt this active transport system, producing a low-flow state that traps pro-inflammatory mediators within the interstitium (90, 91). The process of both inflammatory signaling and biomechanical forces combining to weaken lymphangion contractility provides a mechanistic link between acute immune injury and the chronic edema that fosters progressive ventricular remodeling.

##### Cytokine-mediated contractile suppression (Il-1β, TNF-α)

During inflammation, key inflammatory cytokines blunt the intrinsic contractility of lymphatic muscle cells (91). In particular, interleukin-1β (IL-1β) and tumor necrosis factor-α (TNF-α) are implicated; experimental studies in a rat model demonstrate that IL-1β exposure markedly diminishes the contractility of cardiac lymphatic muscle cells, an effect mediated by the COX-2/prostaglandin E₂ pathway and synergized by TNF-α (91). The study demonstrates that together, these cytokines act as potent lymphatic relaxants, and this mechanism contributes to the progression from acute myocarditis into DCM. This impaired propulsion suggests that even if lymphatic capillaries take up interstitial fluid, the onward transport through pre-collectors and collecting lymphatics is throttled. Indeed, this has been confirmed by a viral myocarditis models (e.g., Theiler's murine virus) that has revealed that proinflammatory cytokine surges cause lymphatic dysfunction and reduced lymph flow out of the heart (91). Cytokine-induced pump failure contributes to the build-up of fluid and inflammatory cells, creating a feedback cycle of edema and sustained inflammation. While cardiac contractions are generally accepted as the main driver of lymph propulsion, a continuous layer of smooth muscle cells has been identified by anti-alpha smooth muscle actin staining in murine collecting cardiac lymphatic vessel walls after postnatal day 30, suggesting its potential role in impaired lymphatic contractility during inflammation (92). However, additional studies are needed to confirm the presence of smooth muscle cells around cardiac lymphatic collecting vessels. Lymphatic vessels in an inflamed myocardium may consequently act as flaccid, non-contractile tubes, unable to efficiently clear accumulating interstitial fluid.

##### Capillary expansion vs. pre-collector slimming can lead to transport mismatch

In addition to direct suppression of contractility, there can be a mismatch in lymphatic transport capacity along the lymphatic network during myocarditis. Initial lymphatic capillaries in the myocardium often become dilated in response to the high interstitial fluid load and inflammatory mediators (93–96). This dilation increases their volume for fluid uptake but may also come at the cost of valve dysfunction and sluggish flow. Meanwhile, the downstream pre-collectors and collecting lymphatics may not proportionally increase their diameter or may even undergo constriction due to inflammatory signaling and external compression (Figure 2). Thus, the lymphatic capillaries are engorged with fluid that cannot be effectively propelled forward because the larger conduits have reduced functional caliber or contractile ability. Supporting this concept, studies have noted that the endogenous lymphangiogenic response produces an abundance of small lymphatic capillaries but a relative paucity of collecting vessels (92). Without sufficient conducting capacity, fluid movement stalls.

Animal studies underscore the impact of impaired lymphatic clearance: in mice with viral myocarditis, lymphatic flow reduction preceded worsening of cardiac inflammation (97). Thus, both intrinsic and extrinsic factors curtail the lymphatic pumping function in myocarditis –cytokines depress the lymphatic pumping, and structural/pressure changes create a bottleneck for lymph transport. Functionally inept lymphatics allow fluid to accumulate unchecked, and thus, myocardial edema persists or worsens. This stagnation also means inflammatory mediators and immune cells are not adequately cleared from the heart tissue, prolonging tissue injury. Indeed, persistent lymphatic drainage failure is proposed as one mechanism by which acute myocarditis transitions into chronic DCM (91). Overall, myocarditis hampers the coordinated lymphatic draining of the heart by both weakening pumping and worsening drainage, ultimately leading to inefficient fluid clearance from the inflamed myocardium.

#### 2.1.3 Lymphangiogenic remodeling

The third sequential blow to the lymphatic system during the progression of myocarditis occurs as the heart attempts to compensate for both structural leakage and pump paralysis through remodeling of the lymphatic network. Inflammatory cues such as VEGF-C stimulate lymphangiogenesis—the formation of new lymphatic vessels—to enhance drainage and restore interstitial fluid homeostasis (90). Yet, this response is somewhat paradoxical in nature: when appropriately regulated, new lymphatics accelerate edema clearance and ameliorate inflammation; however, when inadequate, delayed, or disorganized, they may simply mark ongoing injury or even exacerbate fluid stasis (98) (Figure 2). In this section, we will illustrate signals governing cardiac lymphatic remodeling in viral and autoimmune myocarditis, weighing both the positive and negative contributions of such neovascular growth towards overall myocardial recovery.

##### VEGF-C/VEGFR-3 axis in inflammatory lymphangiogenesis

Myocarditis triggers remodeling of the cardiac lymphatic vasculature, including the growth of new lymphatic vessels as an attempted adaptation to inflammation and edema. Lymphangiogenesis is chiefly governed by the VEGF-C/VEGFR-3 signaling axis: VEGF-C (vascular endothelial growth factor-C) released in the tissue binds to its receptor VEGFR-3 on lymphatic endothelial cells, stimulating them to sprout and form new lymphatic channels (99). Inflammatory conditions strongly upregulate VEGF-C and related factors (such as VEGF-D and IL-7) in many tissues, and the heart is no exception (6). During acute myocarditis, macrophages and other immune cells in the myocardium secrete VEGF-C as part of the innate immune response. A recent study in coxsackievirus B3–induced viral myocarditis showed that cardiac inflammation is accompanied by a surge in lymphatic vessel density, peaking about one week after infection (90). This increase in lymphatics was driven largely by macrophage-derived VEGF-C, as mice depleted of macrophages had blunted lymphangiogenic responses. Notably, blocking VEGF-C signaling through soluble VEGFR-3 traps (which prevent VEGF-C from binding native receptors) led to significantly worse cardiac dysfunction and more severe inflammation in that model. Conversely, therapeutic VEGF-C delivery rescued lymphatic growth and improved cardiac outcomes (90). One important quality of the lymphangiogenic response, however, is its organization and functional sufficiency. If lymphangiogenesis is not structurally complete, it does not serve to effectively relieve congestion. In a mouse model with surgical ablation of collectors, inducing cardiac lymphatic insufficiency, chronic hearts developed edema, inflammation, fibrosis, and diastolic dysfunction despite collateral capillary proliferation, while therapeutic lymphangiogenesis was able to reverse these changes (3). This illustrates that edema persists until a coherent network is rebuilt, and that collector-lacking lymphangiogenesis is functionally insufficient despite vessel growth. Taken together, these findings indicate that inflammation-induced lymphangiogenesis in myocarditis is an adaptive response that can facilitate the resolution of edema and inflammation. By expanding the network of lymphatic vessels, the tissue attempts to drain the excess fluid and immune cell infiltrate more effectively.

## 3 Future directions

Current therapeutics for myocarditis rely on general anti-inflammatory agents, immunosuppression, and antiviral treatments when appropriate, but do not sufficiently target the underlying drivers of myocardial injury and remodeling (100–102). Since lymphatic remodeling is seen in patients with myocarditis, and edema has been shown to amplify the inflammatory response and promote fibrosis, therapeutically enhancing lymphatic function could limit disease progression (93). This can be done through different techniques such as increasing the recruitment of lymphangiogenic factors, indirectly stimulating lymphangiogenesis by recruiting VEGFC-producing immune factors, or augmenting the release of lymphangiogenic factors from cells in the ECM (5, 90, 103). Given the central role of ECM remodeling in both inflammation and fibrosis in myocarditis, therapies that promote lymphatic clearance and tissue repair may also help prevent pathological ECM remodeling. A combined treatment of VEGFC with lymphatic endothelial progenitor cell transplantation was found to reduce cardiac edema and myocardial remodeling and significantly improve cardiac function, with an enhancement of myocardial regeneration following edema clearance (104). Similarly, stem cell therapy using a patch system for endogenous cardiac repair was found to increase cardiac lymphatics and subsequently improve cardiac function in the infarcted myocardium (105). These findings underscore the therapeutic potential of targeting lymphatic regeneration to enhance cardiac repair.

Given the role of impaired lymphatic drainage in inflammation and tissue remodeling, several strategies have been explored to promote lymphangiogenesis to improve cardiac recovery. In mouse models of myocardial infarction, treatment with adrenomedullin or a recombinant form of VEGFC that selectively activates VEGFR3 has led to the resolution of disease-induced edema and inflammatory factors, successfully averting cardiac fibrosis and dysfunction (18, 106). Additionally, the local delivery of VEGFC using a hydrogel was found to improve lymphatic function and decrease infarct scar size in mice subjected to myocardial ischemia (107). Recently, studies in an animal model of viral myocarditis found that the stimulation of cardiac lymphangiogenesis through adeno-associated viral delivery of VEGFC attenuated inflammation and edema (90). Contrary to these findings, a study has shown that blocking VEGFR3 signaling post-myocardial infarction made no significant difference in macrophage counts, cardiac edema, nor cardiac ejection fraction, suggesting that stimulating lymphatic growth may not be an effective approach for recovering cardiac function (108). Through further clarification of the biological role of cardiac lymphangiogenesis, there is promise for therapeutic lymphangiogenesis in reducing cardiac edema, inflammation, and fibrosis, highlighting its potential as an innovative strategy for mitigating the progression of acute myocarditis into chronic structural remodeling associated with DCM (109, 110). The benefits of stimulating lymphangiogenesis are context-dependent and require further investigation to inform the development of targeted interventions capable of influencing disease progression in myocarditis.

To advance cardiac lymphatic therapies, we would need more noninvasive, real-time imaging tools that can help us directly assess therapeutic efficacy. There are currently no biomarkers used clinically to indicate ongoing lymphangiogenesis (111). Current modalities lack the resolution and functional capabilities to visualize cardiac lymphatics, and methods for direct assessment of cardiac lymphatic transport function, such as cardiac lymphangiography, would be too invasive to be feasible in a clinical setting (112). However, the development of these tools would allow for earlier intervention in clearing myocarditis-associated edema and inflammation. So far, T2 mapping is a reliable CMR technique for detecting myocardial edema and, when combined with biomarkers of fibrosis, may help predict the severity of myocardial injury and inform prognosis in myocarditis (73, 113). Future research should investigate the mechanisms and contributing factors that influence the transition from acute myocarditis to chronic inflammatory cardiomyopathy. Additionally, the development of clinical tools, such as prospective registries, biopsy-based risk stratification, and emerging AI-assisted prognostic models, will be essential to identify patients at highest risk for disease progression and guide early intervention (114).
